# Risk of emergency cesarean section when giving birth in Sweden: A nationwide cohort study comparing women born in countries practicing female genital mutilation, with Swedish-born women

**DOI:** 10.1371/journal.pone.0339166

**Published:** 2025-12-17

**Authors:** Bita Eshraghi, Jonas Hermansson, Lena Marions

**Affiliations:** 1 Dept of Clinical Science and Education, Karolinska Institutet, Division of Obstetrics and Gynecology, Södersjukhuset, Stockholm, Sweden; 2 Dept of Research, Angered Hospital, SV-hospital group, Gothenburg, Sweden; Federal Medical Centre Birnin Kudu, NIGERIA

## Abstract

**Introduction:**

The impact of female genital mutilation (FGM) on obstetric outcomes in high-income countries remains unclear and is an area of ongoing research. This nationwide study aimed to examine the risk of emergency cesarean section among primiparous women from countries where FGM is practiced, in comparison to Swedish-born women. Additionally, the study explored whether a confirmed diagnosis of FGM contributes to this potential risk.

**Materials and methods:**

A Swedish nationwide cohort study including primiparous singleton term deliveries (≥37 + 0– < 42 + 0 weeks) during 2014–2020. Data was extracted from the Swedish Medical Birth Register and the National Patient Register. We compared the risk of emergency cesarean section between women born in an FGM-practicing country and women born in Sweden by using multivariable logistic regression. Two subgroup analyses were performed.

**Results:**

Women born in FGM-practicing countries (n = 13 246) had a significantly increased risk of emergency cesarean section (aOR 1.28, 95% CI: 1.21–1.35) compared to Swedish-born women (n = 199 914). Including FGM diagnosis as a covariate did not alter the result. When excluding women born in FGM-practicing countries outside Sub-Saharan Africa the aOR was amplified (aOR 1.58, 95% CI: 1.47–1.69). To further isolate the effect of FGM itself, we restricted the analysis to women born in FGM-practicing countries and compared those with a recorded FGM diagnosis to those without. This analysis showed no significant association between FGM diagnosis and emergency cesarean section (aOR 1.05, 95% CI: 0.90–1.23).

**Conclusions:**

Primiparous women from FGM-practicing countries have an increased risk of emergency CS compared to Swedish-born women, when giving birth in Sweden. The presence of FGM diagnosis did not contribute to this risk. Further research is needed to understand the underlying mechanisms for this to be able to improve obstetric care for migrant populations.

## Introduction

Female genital mutilation (FGM), an ancient cultural ritual mainly practiced in parts of Sub-Saharan Africa, the Middle East, and Asia, impacts more than 230 million girls and women across approximately 30 countries worldwide [1]. FGM includes all procedures that involve partial or total removal of the external female genitalia or other injury to the female genital organs for non-medical reasons [1].

The impact of FGM on obstetric outcomes has been investigated in several studies showing associations with increased risks of cesarean section (CS), instrumental delivery, postpartum hemorrhage, episiotomy, prolonged and difficult labor, obstetric lacerations and, early and neonatal death [2–6]. In a recent study, we found an increased risk for obstetric anal sphincter injury among primiparous women with FGM diagnosis giving birth in Sweden in comparison to women without FGM diagnosis [7].

However, in more recent systematic reviews and meta-analysis, the risk of CS in women with FGM has in fact not shown no be increased [8–10]. Yet, when a subgroup analysis was performed based on parity, Sylla et al. found an increased risk of CS among primiparous women with a history of FGM [9]. Overall, the studies included in these reviews were of low methodological quality and had substantial heterogeneity in study design, settings, population characteristics, and the definition of outcomes. Additionally, most research was conducted in low-resource settings, limiting the generalizability of findings to high-income contexts with different healthcare infrastructures.

As 120 million of the global population are forcibly displaced due to persecution, conflict and human rights violations, the number of migrant women giving birth in receiving countries is continuously rising [11]. Disparities in CS rates have been observed between migrant women and those born in the receiving high-income countries [12]. A recent meta-analysis among migrant in Europe showed that women from Sub-Saharan Africa had higher emergency CS rates than native-born women (OR= 1.64; 95% CI: 1,29–2.08) [13]. Higher CS rates were also found among South and East Asian women. FGM has been proposed to influence the risk of CS, however results are inconsistent and support for causality is lacking [2,8,14–17].

Studies conducted in Western settings have yielded varying and inconclusive results regarding the association between FGM and emergency CS rates [15–19]. Thirty percent of women giving birth in Sweden are born in foreign countries, with two-thirds of these women originating from Africa or Asia [20]. According to the National Board of Health and Welfare, an estimated 68 000 women and girls who have undergone FGM live in Sweden. The highest estimated prevalence of FGM among migrant groups in Sweden is found in women from Somalia, followed by Eritrea, Ethiopia, Egypt, Gambia, and Sudan [21]. Several studies from the Nordic countries have identified women from Sub-Saharan Africa as a high-risk group for emergency CS [22–26], but no previous study has investigated the association between FGM and the risk of emergency CS in Sweden.

There is no clearly suggested causal pathway between FGM and emergency CS. In addition to the proposed limited ability to perform vaginal examinations [16] other contributing factors may include clinical practice patterns, such as healthcare providers’ reluctance to use forceps or vacuum extraction or to perform intrapartum deinfibulation which could lower the threshold opting for cesarean delivery [27]. These factors may contribute to an increased risk of emergency CS among women with FGM.

The objective of this study was to examine whether primiparous women from countries where FGM is practiced are at higher risk of undergoing emergency cesarean section when giving birth in Sweden, compared to women born in Sweden. Given the limited evidence supporting a direct link between FGM and emergency cesarean section, this study further aimed to explore whether FGM itself plays a significant role in the potential increased risk of cesarean delivery, or if it is better explained by a combination of other factors associated with women born in FGM-practicing countries, particularly in Sub-Saharan Africa.

In Sweden, maternity care is universally accessible and publicly funded. Identifying groups at increased risk for obstetric complications is crucial for improving equity in maternal healthcare. Understanding potential disparities in emergency CS rates between migrant women from FGM-practicing countries compared to Swedish born women is important to provide optimal obstetric care for the whole Swedish population.

## Materials and methods

The study population consisted of 329 140 primiparous women with singleton deliveries giving birth in Sweden during 2014−2020. The exposure was defined as being born in an FGM-practicing country, with the comparison group consisting of individuals born in Sweden. The outcome was emergency CS. We excluded pre- and post-term deliveries (<37 weeks and ≥42 weeks of gestation), women born in non-FGM-practicing countries other than Sweden, planned CS and women with unknown country of birth. Data for this nationwide register-based cohort study was obtained from the Swedish Medical Birth Register (MBR). The register compiles information prospectively collected from standardized antenatal, obstetric, and neonatal checkboxes across all antenatal clinics and hospitals in Sweden. It also includes medical diagnoses classified according to the International Classification of Diseases 10 (ICD-10) and Classification of procedure codes (KVÅ). The MBR is validated and includes demographic data, reproductive history, maternal health, as well as pregnancy- and neonatal outcomes for over 98% of all births in Sweden [28]. Data from the National Patient Register (NPR) was also used in this study to identify any FGM diagnosis that was not included in the MBR. Both registers are maintained by the National Board of Health and Welfare in Sweden. Data on maternal and neonatal characteristics including maternal age (year), Body Mass Index (BMI) (kg/m^2^), height (cm), length of gestation (weeks) and infant birthweight (gram) were collected. Small for gestational age (SGA) and large for gestational age (LGA) were defined as birthweight below the 10th percentile and above the 90th percentile, respectively, according to Swedish growth standards.

Cases of emergency CS were identified by the ICD-10 code O82.1 (emergency CS). If the woman had the ICD-codes O82.2 (CS with hysterectomy), O82.8 (CS without medical indication), or O82.9 (CS, unspecified) they also needed to have the procedure code ZXD00 (acute surgery) to be considered emergency CS. The codes were combined with the information from the MBR checkboxes to further distinguish cases of emergency CS from planned CS.

FGM-practicing countries were defined in accordance with those reported by United Nations Children’s Fund (UNICEF) [29]. Diagnosis of FGM was defined as one or more of three specific ICD-10 codes for FGM (Z917, Z907, O347A), in either the MBR or the NPR. To prevent misclassification of cases with Z907 related to vulvectomy due to vulvar precancerous or cancerous conditions, we excluded women who had received a Z907 code along with a diagnosis related to vulvar cancer (C510, C511, C512, C518, C519, D071, Z854A). No information was available from MBR or NPR regarding the specific type of FGM.

Chi-square analysis was performed to assess differences regarding baseline characteristics and maternal country of birth (Sweden vs FGM-practicing countries). Multivariable logistic regression was used to estimate the odds ratio (OR) with 95% confidence interval (CI), to assess the association between being born in an FGM-practicing country and emergency CS. Covariates included in multivariable analyses were decided a priori based on previous knowledge and were added stepwise. In the adjusted regression models, we first included maternal age followed by BMI and height. Next, we added infant birthweight, and induction of labor. Finally, we included the diagnosis of FGM. Covariates in the multivariable logistic regression were categorized as follows: maternal age (<25, 25–34, ≥ 35 years), BMI (<18.5, 18.5–24.9, 25.0–29.9, ≥ 30 kg/m²), maternal height (<150, 150–159, 160–169, ≥ 170 cm), and infant birthweight (<2500, 2500–2999, 3000–3499, 3500–3999, 4000–4499, ≥ 4500 g). In all regression models, the following categories were used as references: maternal age 25–34 years, BMI 18.5–24.9 kg/m², maternal height 160–169 cm, and infant birthweight 3000–3499 g. Swedish-born women were used as the reference group for maternal country of birth. We performed complete case analysis. A p-value of 0.05 or less was considered statistically significant.

To assess the robustness of our results we performed two subgroup analyses. The main exposure group contains a large proportion of women from Iraq, out of which predominantly Kurdish women are affected by FGM who could not be distinguished from unexposed Iraqi women. Given the above, and that originating from Sub-Saharan Africa may imply other risk factors of importance for health outcomes, we chose to exclude women born in FGM-countries outside of Sub-Saharan Africa in a subgroup analysis.

First, we exclusively included women born in FGM-practicing countries in Sub-Saharan Africa in comparison with women born in Sweden, thus excluding women born in Iraq, Egypt, Indonesia, Yemen and Maldives. Secondly, we restricted the analysis to women born in FGM-practicing countries and evaluated whether an FGM diagnosis was associated with increased risk of emergency CS. This contrasts with the main cohort analysis, which compared women born in FGM-practicing countries with those born in Sweden.

Statistical analyzes were performed using IBM SPSS Statistics software version 29 for PC.

Ethical permission for this study was obtained from the Swedish Ethical Review Authority and its regional predecessor in Stockholm (D nr (2018/1297-31/3), (2019−02323), (2021-07009-02), (2024-06881-02)). The data was analyzed anonymously, oral or written consent was not required. The authors had no access to information that could identify individual participants. The data was accessed for research purposes on the 10^th^ of February 2023.

## Results

**Fig 1:**
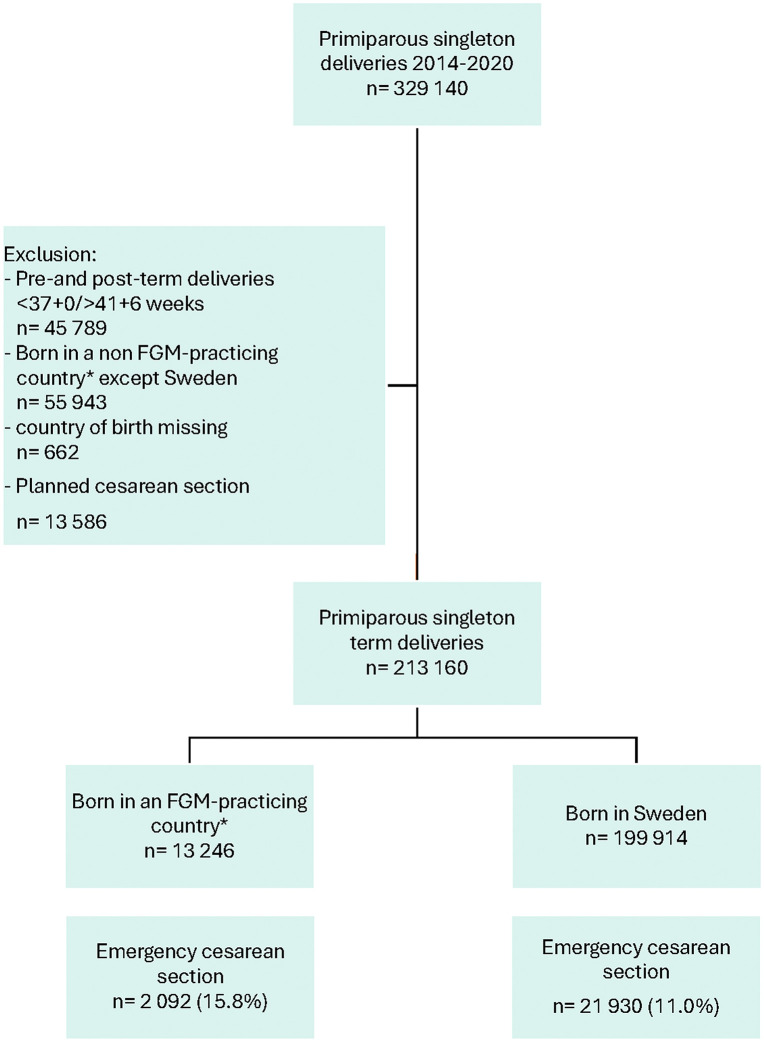
Flowchart of participants in this study based on data from the Swedish Medical Birth Register (MBR). *FGM-practicing country according to the United Nations Children’s Fund (UNICEF).

**Fig 2:**
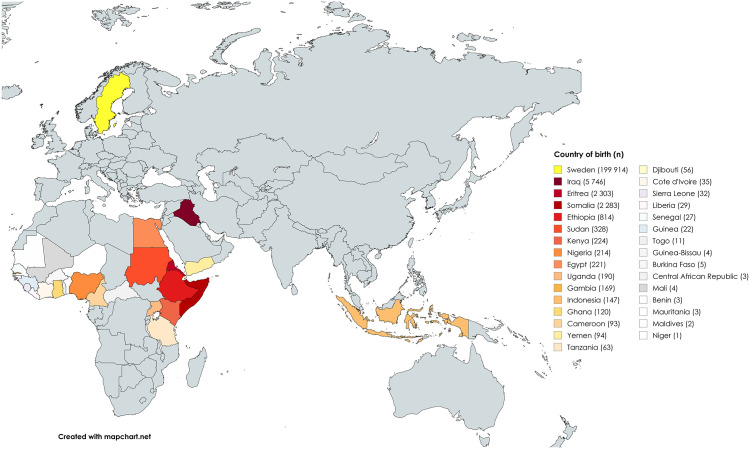
Participants’ country of birth.

The final study population consisted of 213 160 women, of which 13 246 were born in an FGM-practicing country and 199 914 were born in Sweden (Fig 1). Country of birth is shown in Fig 2. In the exposure group, 43% (n = 5 746) of women were born in Iraq, 17% (n = 2 303) in Eritrea, 17% (n = 2 283) in Somalia and 6% (n = 814) in Ethiopia. Together, these countries represent the majority of the exposure group. FGM diagnosis was most present in women from Somalia 38% (n = 874), Sudan 26% (n = 85), Djibouti 27% (n = 15), Eritrea 17% (n = 397) and Ethiopia 15% (n = 122). In total, 11.9% (n = 1 573) of women in the exposure group had a confirmed FGM diagnosis.

Maternal and infant characteristics are shown in Table 1. Mode of delivery, neonatal outcomes and maternal medical conditions are shown in Table 2. In the study population, the overall rate of emergency CS was 11.3%. A larger proportion of women born in FGM-practicing countries underwent emergency CS, induction of labor, instrumental delivery, had infants born with APGAR-score <7 at 5 minutes and intrauterine fetal death (IUFD) compared to women born in Sweden (Table 2). Conditions present or indication for emergency CS by country of birth are shown in S1 Table. Fetal distress was present or an indication for emergency CS in 47.6% among women from FGM-practicing countries in comparison to 36.0% among women born in Sweden. Women from FGM-practicing countries who underwent emergency CS had similar proportion of labor dystocia or failure to progress compared to women born in Sweden (S1). Maternal and obstetric characteristics among women delivered with emergency CS comparing women born in FGM-practicing countries and women born in Sweden are shown in S2 Table.

**Table 1:** Maternal and infant characteristics of primiparous women with singleton term delivery.

		Born in FGM-practicing country		Born in Sweden	
		(n = 13 246)		(n = 199 914)	
		n	%	n	%
Maternal age, years	<25	4 813	36.3	36 669	18.3
	25-34	7 331	55.3	142 044	71.1
	≥35	1 102	8.3	21 201	10.6
Maternal height, cm	<150	152	1.2	163	0.1
	150-159	4 447	34.9	16 848	8.8
	160-169	6 948	54.5	103 397	53.8
	≥170	1 211	9.5	71 738	37.3
Missing		488	3.7	7 768	3.9
Maternal BMI, kg/m2	< 18.5	856	6.8	4 429	2.3
	18.5-24.9	7 055	56.2	114 696	60.7
	25.0-29.9	3 223	25.7	45 801	24.3
	≥30	1 422	11.3	23 912	12.7
Missing		690	5.2	11 076	5.5
Birthweight, g	< 2500	366	2.8	2 513	1.3
	2500-2999	2 637	19.9	22 120	11.1
	3000-3499	5 814	43.9	74 512	37.3
	3500-3999	3 511	26.5	71 947	36.0
	4000-4499	812	6.1	24 631	12.3
	≥4500	101	0.8	4 091	2.0
Missing		5	<0.0	100	0.1
FGM diagnosis		1 573	11.9	19	<0.0

**Table 2:** Medical diagnosis, mode of delivery and neonatal outcomes among primiparous women with singleton term delivery.

	Born in FGM-practicing country (n = 13 246)		Born in Sweden	
			(n = 199 914)	
	n	%	n	%
Gestational diabetes mellitus	628	4.7	43 23	2.2
Diabetes mellitus	90	0.7	1 556	0.8
Preeclampsia/Eclampsia	472	3.6	9 118	4.6
Essential hypertension	44	0.3	1 248	0.6
Emergency cesarean section	2 092	15.8	21 930	11.0
Instrumental delivery	1 493	11.3	20 700	10.4
Vaginal delivery	9 661	72.9	157 284	78.7
Induction of labor	3 152	23.8	40 089	20.1
Epidural	6 973	52.6	115 822	57.9
Apgar <7 at 5 min	346	2.6	3 015	1.5
IUFD	33	0.3	269	0.1
SGA	846	6.4	5 009	2.5
LGA	108	0.8	3 911	2.0

IUFD: Intrauterine fetal death, SGA: Small for gestational age, LGA: Large for gestational age.

The multivariable logistic regression analysis showed a significant association between women born in a country where FGM is practiced and emergency CS (aOR 1.28, 95% CI: 1.21–1.35). When FGM-diagnosis was included as a covariate in the model, the result was unaltered (aOR 1.27, 95% CI: 1.19–1.34) (Table 3).

**Table 3:** Crude and adjusted odds ratios (OR) and 95% confidence intervals (CI) for emergency cesarean section comparing women born in FGM-practicing countries (n = 13 246) with women born in Sweden (n = 199 914) (born in Sweden as the reference).

	OR (95% CI)
Crude	1.52 (1.45-1.60)
Model 1: Adjusted for maternal age	1.68 (1.60-1.76)
Model 2: Adjusted for maternal age and BMI	1.70 (1.61-1.79)
Model 3: Adjusted for maternal age, BMI and height	1.27 (1.20-1.34)
Model 4: Adjusted for maternal age, BMI, height, infant birthweight and induction of labor	1.28 (1.21-1.35)
Model 5: Adjusted for maternal age, height, BMI, infant birthweight, induction of labor and FGM diagnosis	1.27 (1.19-1.34)

In the first subgroup analysis, we excluded women born in Iraq, Egypt, Indonesia, Yemen and Maldives (n = 6 210), thus exclusively comparing women born in an FGM-practicing country in Sub-Saharan Africa (n = 7 036) with women born in Sweden, and the association of emergency CS (Table 4). The adjusted OR for emergency CS in this group was increased (aOR 1.58, 95% CI: 1.47–1.69) compared to the main analysis where women born in all FGM-practicing countries were included (aOR 1.28, 95% CI: 1.21–1.35) (Table 4 and Table 3). When FGM-diagnosis was included as a covariate in the model, the result was unaltered (aOR 1.63, 95% CI: 1.52–1.77).

**Table 4:** Crude and adjusted odds ratios (OR) and 95% confidence intervals (CI) for emergency cesarean section. Subgroup analysis comparing women born in FGM-practicing countries in Sub-Saharan Africa (n = 7 036) and Sweden (n = 199 914) (born in Sweden as the reference).

	OR (95% CI)
Crude:	1.85 (1.74-1.97)
Model 1: Adjusted for maternal age	2.03 (1.91-2.17)
Model 2: Adjusted for maternal age and BMI	2.13 (2.00-2.27)
Model 3: Adjusted for maternal age, BMI and height	1.65 (1.54-1.76)
Model 4: Adjusted for maternal age, height, BMI, fetal birthweight and induction of labor	1.58 (1.47-1.69)
Model 5: Adjusted for maternal age, height, BMI, fetal birthweight, induction of labor and FGM diagnosis	1.63 (1.52-1.77)

In the second subgroup analysis, we examined the association between having or not having an FGM diagnosis and emergency CS, among women born in FGM-practicing countries. We found no significant association between FGM diagnosis and emergency CS (Table 5).

**Table 5:** Subgroup analysis comparing women born in FGM-practicing countries with and without diagnosis of FGM. Crude and adjusted odds ratios (OR) and 95% confidence intervals (CI) for emergency cesarean section.

			Emergency cesarean section	
	All (n = 13 246)	n = 2 092		
			cOR (95% CI)	aOR (95% CI)
FGM diagnosis				
No	11 673	15.8%	1	1
Yes	1 573	15.8%	1.00 (0.87–1.16)	1.05 (0.90-1.23)

cOR- Crude odds ratio; aOR= Adjusted odds ratio.

Adjusted for maternal age, BMI and height, fetal birthweight and induction of labor.

## Discussion

In our study among primiparous women giving birth in Sweden between 2014–2020, we found that women originating from a country where FGM is practiced had an increased risk of emergency CS compared to women born in Sweden. Previous studies exploring the risk of emergency CS among migrant groups in receiving countries have also shown increased risk [12,22,23,26,30], however, the population in those studies generally were selected from a specific country or region, such as Somalia or Sub-Saharan Africa. To our knowledge, this is the first nationwide study from a high-income country that investigates the association between being born in an FGM-practicing country and giving birth by emergency CS.

Although a clearly defined causal pathway between FGM and emergency CS remains uncertain, several studies have reported an association between FGM and labor complications, particularly prolonged or arrested labor [27,31,32]. While prolonged labor may raise the likelihood of emergency CS, FGM does not appear to be a probable cause. In fact, a Swedish study found that infibulated women had a shorter second stage of labor and a lower risk of prolonged labor compared to women without FGM [33]. From a physiological perspective, it is not plausible that scarring of the external genitalia, as seen in FGM, would significantly impede the progress of labor [31,34]. Furthermore, according to international guidelines, deinfibulation is recommended in cases of FGM type 3 (infibulation) as part of obstetric management to avoid obstruction at the final stage of delivery [6,35]. It has also been proposed that there is a dose-response relationship between the degree of FGM and the risk of obstetric complications [2]. However, two recent studies that examined the risk of emergency CS in relation to FGM type, could not show an increased risk for infibulated women [31,36]. In our study the presence of FGM diagnosis did not increase the risk for emergency CS.

Previous European studies have shown that migrants from countries in Sub-Saharan Africa have an increased risk of emergency CS compared to women born in the receiving country [22–25,30,37]. FGM has sometimes been suggested as one of several possible factors contributing to this increased risk. Given the lack of convincing evidence linking emergency CS with FGM, we aimed to further investigate whether FGM plays a major role in the increased risk of CS, or if this risk is due to a combination of other factors present in women born in countries where FGM is practiced, particularly in Sub-Saharan Africa. For this reason, we chose to compare women born in a country where FGM is practiced with women born in Sweden, adjusting for FGM diagnosis as a covariate in the regression model. Although we could show an increased risk for emergency CS among women from FGM-practicing countries, our approach allowed us to conclude that FGM diagnosis did not play a significant role in the association between country of birth and emergency cesarean section.

Previous studies suggest that the mechanisms leading to cesarean delivery often are complex and likely to involve a combination of factors that affect health [12].

The most common risk factors associated with CS among migrant populations include: communication barriers, poor maternal health, low socioeconomic status, gestational diabetes mellitus, high BMI, feto-pelvic disproportion and quality of care [12]. While migrants at large may face some similar challenges in receiving countries, there are also differences in many aspects depending on region of origin. Black African migrants may in addition more often experience racial discrimination in society as well as within the healthcare system, which could have a negative impact on health [38,39]. Migrants from Sub-Saharan Africa, which includes a majority of the FGM-practicing countries, have shown to be at higher risk of emergency CS after migration to high-income countries compared to women born in receiving countries [12,13]. In Sweden, several studies have reported suboptimal care and increased risk of maternal and perinatal mortality among women born in Sub-Saharan Africa in comparison to women born in Sweden [40–42].

When we analyzed the exposure group, women born in FGM-practicing countries, we observed that a large proportion originated from Iraq (43%), a country with more than 45 million inhabitants and an estimated prevalence of FGM of 7.4%. However, the practice is primarily prevalent in the Iraqi Kurdistan Region, an autonomous region in northern Iraq with approximately six million inhabitants [43]. In this region, approximately 37.5% of women have been subjected to FGM, a significant contrast to the 0.4% prevalence observed in the central and southern parts of Iraq. In our study population, we could not distinguish women from the Kurdish Region of Iraq, however it is reasonable to believe that most women originating from Iraq had not been subjected to FGM. Given the large proportion of Iraqi women included in the exposure group, and that originating from Sub-Saharan Africa may imply other risk factors of importance for health outcome, we chose to exclude women born in FGM-countries outside of Sub-Saharan Africa in a subgroup analysis (Table 4). This approach further offers valuable comparison with previous studies of health outcomes among migrants from this region. Our analysis revealed that the risk of emergency CS was further elevated when exclusively analyzing women born in Sub-Saharan Africa in comparison with that for women born in all FGM-practicing countries, in relation to women born in Sweden. The diluting effect on the outcome when including women born in FGM-practicing countries outside of Sub-Saharan Africa may be caused by differences in sociocultural background, as maternal characteristics is similar among women in the entire exposure group.

The baseline characteristics of women originating from FGM-practicing countries showed that they were younger compared with to women born in Sweden, and they more seldom gave birth to infants weighing over 4000 grams. While the combination of younger age and a lower incidence of macrosomia may reduce the need for emergency CS, the higher proportion of women shorter than 160 cm in this group constitutes a potential risk factor as short stature can be associated with a higher risk of cephalopelvic disproportion [44]. This factor, closely tied to maternal origin, could lead to challenges during labor that may counterbalance the protective factors. However, by doing a stepwise analysis we were able to analyze the role of maternal height separately. When maternal height was included in the adjusted model, the risk of emergency CS among women from FGM-practicing countries or Sub-Saharan Africa decreased but still remained significantly elevated compared to women born in Sweden. This suggests that part of the observed disparity in the risk of emergency CS can be attributed to differences in maternal height. Further explanation may be found in other aspects of intrapartum care, such as inadequate support and information, timing of interventions, healthcare providers’ attitudes and preconceived ideas of obstetric interventions for migrant women, communication barriers and discrimination.

Our findings can raise awareness about the increased risk of emergency CS among migrant women from FGM-practicing countries, in particular Sub-Saharan Africa, independent of FGM status. Clinicians should therefore avoid attributing these higher CS rates solely to FGM. This can contribute to shifting the focus to other potential contributing factors that elevate the risk for emergency CS, leading to more targeted strategies aimed at optimizing maternal outcomes and potentially reducing the incidence of emergency cesarean sections for these women.

Major strengths of this study include the use of a large population-based registry with validated high-quality data inputs and a relatively low percentage of missing data. Sweden is an appropriate setting for studies such as ours, because all births occur within a public health system offering maternal care, free of charge. A majority of FGM-practicing countries are represented in the material. Due to limitations in data availability, we had no information on socioeconomic status or language skills, which might contribute to differences between the groups. Also, information on the indication of cesarean delivery is not available in the MBR. Lastly, there is probably an under-reporting of FGM diagnosis in Swedish registers considering the incidence of FGM reported by UNICEF. At the time of data collection, there was no national guideline in Sweden for the management of women with FGM or from FGM-practicing countries; such guidance has since been developed and became operational in 2024. This should be considered when interpreting the findings in relation to current clinical practice.

## Conclusion

We conclude that primiparous women from FGM-practicing countries have an increased risk of emergency CS compared to Swedish-born women, when giving birth in Sweden. The presence of FGM diagnosis did not contribute to this risk. We believe that there are other factors than the presence of FGM diagnosis that increase the risk of emergency cesarean section for women born in FGM practicing countries, when giving birth in Sweden.

## Supporting information

S1 Table Conditions present and/or indications for emergency cesarean section by country of birth. (DOCX)

S2 Table Maternal and obstetric characteristics among primiparous women delivered with emergency cesarean section. (DOCX)
