# Supplementary material for: Risk of emergency cesarean section when giving birth in Sweden: A nationwide cohort study comparing women born in countries practicing female genital mutilation, with Swedish-born women
Source: PLoS One. 2025 Dec 17;20(12):e0339166. doi: 10.1371/journal.pone.0339166 (PMC12711012; doi:10.1371/journal.pone.0339166)
Supplement: S1 Table — (DOCX) [file pone.0339166.s001.docx]

|  | Emergency CS (n=24 022) | | | |
| --- | --- | --- | --- | --- |
|  | Born in FGM-practicing country (n= 2092) | | Born in Sweden (n= 21 930) | |
|  |  |  |  |  |
|  | n | % | n | % |
| Fetal distress | 966 | 47.6 | 7 899 | 36.0 |
| Failure to progress/ inadequate contractions | 860 | 41.1 | 9 523 | 43.4 |
| Failed attempted instrumental delivery | 143 | 6.8 | 1 147 | 5.2 |
| Preeclampsia/ eclampsia/ HELLP | 180 | 8.6 | 2 434 | 11.1 |
|  |  |  |  |  |
| Breech | 88 | 4.9 | 1 992 | 10.2 |
| Other malpresentation | 163 | 7.4 | 1 828 | 8.3 |
| Disproportion between pelvis and fetus | 92 | 4.2 | 752 | 3.4 |
|  |  |  |  |  |
| Umbilical cord complications | 22 | 1.0 | 211 | 1.0 |
| Placenta ablatio | 29 | 1.3 | 315 | 1.4 |
