# Supplementary material for: Risk of emergency cesarean section when giving birth in Sweden: A nationwide cohort study comparing women born in countries practicing female genital mutilation, with Swedish-born women
Source: PLoS One. 2025 Dec 17;20(12):e0339166. doi: 10.1371/journal.pone.0339166 (PMC12711012; doi:10.1371/journal.pone.0339166)
Supplement: S2 Table — (DOCX) [file pone.0339166.s002.docx]

|  |  | Emergency cesarean section | | | |
| --- | --- | --- | --- | --- | --- |
|  |  | Born in FGM-practicing country | | Born in Sweden | |
|  |  | (n= 2 092) | | (n= 21 930) | |
| Maternal age, years | |  |  |  |  |
| <25 | | 156 | 11.7% | 2 621 | 7.1% |
| 25–34 | | 1 212 | 16.5% | 14 932 | 10.5% |
| ≥35 | | 317 | 28.8% | 4 377 | 20.6% |
|  | |  |  |  |  |
| Maternal height, cm | |  |  |  |  |
| <150 | | 56 | 36.8% | 53 | 32.5% |
| 150-159 | | 908 | 20.4% | 3 214 | 19.1% |
| 160-169 | | 946 | 13.6% | 12 083 | 11.7% |
| ≥170 | | 109 | 9% | 5 801 | 8.1% |
|  | |  |  |  |  |
|  | |  |  |  |  |
| Maternal BMI, kg/m2 | |  |  |  |  |
| <18,5 | | 82 | 9.6% | 283 | 6.4% |
| 18.5-24.9 | | 933 | 13.2% | 10 240 | 8.9% |
| 25-29.9 | | 598 | 18.6% | 6 038 | 13.2% |
| ≥30 | | 375 | 26.4% | 4222 | 17.7% |
|  | |  |  |  |  |
|  | |  |  |  |  |
| Birthweight, g | |  |  |  |  |
| < 2500 | | 92 | 25.1% | 681 | 27.1% |
| 2501-3000 | | 739 | 12.7% | 2 496 | 11.3% |
| 3001-3500 | | 346 | 13.1% | 6 237 | 8.4% |
| 3501-4000 | | 623 | 17.7% | 7 143 | 9.9% |
| 4001-4500 | | 237 | 29.2% | 4 132 | 16.8% |
| >4500 | | 53 | 52.5% | 1 224 | 29.9% |
|  | |  |  |  |  |
|  | |  |  |  |  |
| Induction of labor | |  |  |  |  |
| No | | 1 094 | 10.8% | 13 396 | 8.4% |
| Yes | | 998 | 31,70% | 8 534 | 21.3% |
|  | |  |  |  |  |
| FGM diagnosis | |  |  |  |  |
| No | | 1 843 | 15.8% | 21 927 | 11% |
| Yes | | 249 | 15.8% | 3 | 15.8% |
